# Developmental defects of enamel in primary teeth and association with early life course events: a study of 6–36 month old children in Manyara, Tanzania

**DOI:** 10.1186/1472-6831-13-21

**Published:** 2013-05-14

**Authors:** Ray Masumo, Asgeir Bårdsen, Anne Nordrehaug Åstrøm

**Affiliations:** 1 Department of Clinical Dentistry, University of Bergen, Bergen, Norway; 2 Centre for International Health, University of Bergen, Bergen, Norway; 3 Muhimbili University of Health and Allied Sciences, Dar Es Salaam, Tanzania

## Abstract

**Background:**

Children with low birth weight show an increased prevalence of developmental defects of enamel in the primary dentition that subsequently may predispose to early childhood caries (ECC).

Focusing 6–36 months old, the purpose of this study was to assess the frequency of enamel defects in the primary dentition and identify influences of early life course factors; socio-demographics, birth weight, child’s early illness episodes and mothers’ perceived size of the child at birth, whilst controlling for more recent life course events in terms of current breastfeeding and oral hygiene.

**Methods:**

A cross-sectional study was conducted in the high fluoride area of Manyara, northern Tanzania including 1221 child-mother pairs who attended Reproductive and Child Health (RCH) clinics for immunization and/or growth monitoring. After the primary caregivers had completed face to face interviews at the health care facility, children underwent oral clinical examination whereby ECC and developmental defects of enamel were recorded using field criteria. All erupted teeth were examined and the enamel defects were assessed on buccal surfaces according to the modified DDE Index.

**Results:**

The prevalence of enamel defects was 33.3%. Diffuse opacities were the most common defects identified (23.1%), followed by hypoplasia (7.6%) and demarcated opacities (5.0%). The most frequently affected teeth were the upper central incisors (29.0% - 30.5%), whereas lower central incisors (4.3% to 4.5%) were least frequently affected. Multiple logistic regression analysis, adjusting for confounding the factors revealed that having normal birth weight (equal or more than 2500 g) associated with lower odds of having enamel hypoplasia [OR 0.2 (95% CI 0.1-0.7)]. No statistically significant association occurred between birth weight and diffuse opacities, demarcated opacities or combined DDE.

**Conclusion:**

Children with the history of low birth weight were more likely than their normal birth weight counterparts to present with enamel hypoplasia. In view of the frequent occurrence of enamel defects and the fact that hypoplasia may constitute a risk factor for future ECC, enamel defects should be included as a dental health indicator in epidemiological studies of children in northern Tanzania.

## Background

Developmental defects of enamel (DDE) in the primary dentition are visible deviations from the normal translucent appearance of tooth enamel resulting from damage of the enamel organ during amelogenesis [1]. Clinically DDE can be classified into three types; demarcated opacity, diffuse opacity and hypoplasia [1]. Opacity is a hypo-mineralization defect involving alteration in the translucency of enamel. Hypoplasia is a quantitative defect associated with a reduced thickness of enamel and appears as grooves or pits [1,2]. These enamel defects can have a significant impact on esthetics, tooth sensitivity and occlusal function [3-5]. Moreover, enamel hypoplasia has been described as one predisposing factor for ECC and erosion [6-9]. Thus, primary dentition with incomplete enamel calcification on pits and fissures provides suitable sites for the adhesion and colonization of cariogenic bacteria. Consequently, ECC will develop more rapidly on the altered tooth surfaces [9].

Studies considering the prevalence and covariates of DDE vary considerably with respect to characteristics of the populations investigated, measurement aspects and study design utilized [10]. This should be taken into consideration when comparing findings of the various research reports. Epidemiological studies have suggested an increase in the frequency of occurrence of DDE in all populations, thus underlining their clinical significance and public health importance [4,11]. Among healthy children in developed countries the prevalence of DDE in primary teeth has been reported to range between 24% and 49% [11,12]. Robles et al. [12] reported on a prevalence of enamel defects amounting to 40.2% in primary teeth of Spanish children 3–12 years of age. Seow et al. [4] reported on a prevalence of 25% in a low-fluoridated community in Australia. Slayton et al. [11] reported a prevalence of hypoplasia of 6% and a prevalence of isolated opacities of 27% among 4–5 year olds in Iowa (USA). Similar findings have been reported from developing countries. A recent study by Correa-Faria et al. [13] revealed a prevalence of DDE of 30% among 3–5 year olds in Brazil. Matee et al. [7] investigated 1–4 year olds in different regions of Tanzania and identified a frequency of occurrence that varied from 2.7% to 11%.

The amelogenesis of primary teeth starts in the 15^th^ gestational week and completes its development 12 months after birth (second deciduous molar) [14,15]. The risk of DDE is related to social factors [16-18], nutritional problems [17,18], excessive exposure to fluoride and infectious diseases [16,17,19] occurring during the pre- and post natal period of amelogenesis. However, the exact mechanism and etiological factors are not fully understood [15]. Previous studies have shown that maternal ingestion of chemicals such as fluorides, tetracycline and thalidomide are associated with higher prevalence of DDE [16,20]. In a longitudinal study of enamel hypoplasia and life course events of 12–36 months old Brazilian children, under nutrition and childhood infections during the period of tooth development were associated with enamel defects in socioeconomically underprivileged communities [18]. Among the most prevalent oral alterations in prematurely borne (i.e. a new borne of less than 37 weeks gestation) and low birth weight children (i.e. less than 2500 gram at birth) are hypoplasia and opacities in the dental enamel [13,21-23]. In a study of 2–6 years old Saudi boys, malnutrition, low birth weight, childhood illness and brushing child’s teeth were identified as risk indicators of enamel defects [17]. Other studies have also identified low birth weight as a risk indicator of enamel defects [22].

### Purpose

Although evidence suggests that DDE are important risk factors for dental caries in the primary as well as the permanent dentition, population based studies considering its prevalence and early life course determinants are scarce, especially in economically less privileged samples of developing countries. Focusing 6–36 months old in Manyara region, Tanzania, the purpose of this study was to assess the frequency of enamel defects in the primary dentition and identify influence from early life course factors; socio-demographics, birth weight, child’s early illness episodes and mothers’ perceived size of the child at birth, whilst controlling for more recent life course events in terms of current breastfeeding and oral hygiene.

## Methods

The study population comprised all child- caretaker pairs attending the Reproductive and Child Health (RCH) care facilities in Haydom Lutheran Hospital (HLH) and its 20 mobile outreach community service sites in Mbulu, Hanang and Babati districts of Manyara, Northern Tanzania, from August 2010 to January 2011. The community outreach posts are not health facilities but may be in any building available in the respective villages. According to the 2002 population and housing census in Tanzania, the HLH RCH outreach programme covered 6 out of 54 villages in Hanang, 3 out of 81 villages in Babati and 12 out of 70 villages in Mbulu, serving respectively, 4790, 1538 and 7910 children below 5 years of age [24]. During the project period, RCH outreach posts were visited 3–5 times on a rotating basis, recruiting 10–14 caretaker-child pairs per visit. All caregiver-child pairs who were resident in the catchment areas of the RCH posts and who satisfied the inclusion criteria of being a mother or primary caregiver of children aged 6–36 months attending for immunization and/or growth monitoring during the survey period, were invited to participate in the study. Mothers were the primary target respondents (99% of the respondents), but in case of mothers’ absence, the primary caregiver was recruited. Out of 1250 child/caregiver pairs approached, 1221 agreed to participate (total response rate 97.7%). A sample size (n = 1221) of this magnitude is sufficient to the pre-calculated sample size of 810 caregiver-child pairs, assuming a prevalence of early childhood caries, ECC, of 50%, a margin error of 5%, confidence level of 95%, a power of 90% and an assumed design effect of 2. Another 5% was added to the sample size to account for- non responses. Permission was granted by the Medical research Coordinating Committee of Ministry of Health and Social Welfare in Tanzania Research (NIMR/HQ/R.8a/Vol.IX/978) and the Ethical research Committee in Norway (REK VEST). Informed written consent was obtained from participating caregivers in both recruitment sites. When the caregivers could not read and write verbal consents were obtained.

### Interviews

An interview schedule was constructed in English and translated into Kiswahili, the main language in Manyara. Kiswahili is the national language in Tanzania spoken proficiently by almost 95% of the population. The interview schedule was translated in several steps; from English into Kiswahili by bi-lingual Kiswahili/English professionals, and then back translated to English by independent translators. Project professionals in the field reviewed the interview schedule for semantic, experiential and conceptual equivalence to the original version. Sensitivity to culture and selection of appropriate words were considered. The interview schedule was piloted and administered in face to face interviews with primary caretakers before their children underwent a full mouth oral clinical examination.

A theoretical model adapted from the work of Zhou et al. [8], guided the selection of life course explanatory variables and the multivariable analyses. According to its propositions; early life course determinants such as socioeconomic background, developmental characteristics at birth, feeding habits and oral hygiene related characteristics would contribute to the developmental of ECC. Assuming that enamel defects may be important risk factors for the development of ECC, ECC and enamel defects could share those early life course determinants.

Socio-demographic and socio-economic characteristics were assessed in terms of age and sex of the child and caregiver, level of mother’s education and household index. Primary caregiver’s age was recorded in years and a dummy variable was constructed as; (0) ≤ 24 years old, (1) ≥ 25 years old. Mother’s education was assessed by asking; “What is the highest level of school you have attended?” Responses were given as (0) No formal education, (1) Did not complete primary school, (2) Completed primary school, (3) Secondary, (4) Completed Secondary, (5) College/University. A dummy variable was constructed 0= lower education (including the original categories 0 and 1) and 1= at least primary education (including the original categories 2, 3, 4 and 5). Family wealth was assessed as an indicator of socio-economic status according to a standard approach in equity analysis [25]. Durable household assets indicative of family wealth (i.e. radio, television, telephone, refrigerator, lantern, cupboard, bicycle, motor cycle, car, boat) were recorded as (0) not available and/or not in working condition or (1) available and in working condition. These assets were analyzed using principal components analysis (PCA). The first component resulting from this analysis was used to categorize households into four approximate quartiles of wealth ranging from the 1^st^ quartile (least poor) to the 4^th^ quartile (poorest).

Early childhood developmental factors were assessed in terms of mother’s perception of child’s size at birth, actual birth weight and childhood illness episodes. Perceived child size at birth was categorized in terms of (0) smaller than average (1) average (2) larger than average?” Birth weight of each child was obtained from birth certificate or immunization card and a dummy variable was constructed according to the World Health Organization (WHO) definition [26] as; (0) low birth weight (<2599 g) and, (1) normal birth weight (≥2500 g). Childhood illness in terms of episodes of infection was assessed by asking mothers “Has (Name) had episodes of ill with fever, cough, and diarrhea since birth?” Responses were given as (1) No and (2) Yes. A sum score was constructed (range 3–6) and dichotomised based on the median (score 5) split into 0= few episodes and 1= many episodes. More recent life course events in terms of current breastfeeding was assessed by asking mothers “Do you breastfeed (Name)?” and responses was (1) yes and (0) No.

### Clinical examination

Clinical oral examinations were conducted by a trained and calibrated dentist (RM), whereas trained assistants recorded the observations. Calibration exercises for the examiner with respect to early childhood caries were carried out according to the guidelines published by British Association of the Study of Community Dentistry (BASCD) [27]. Children were examined in knee to knee position using a dental mirror and natural light. Current oral hygiene in terms of visible plaque in the upper anterior teeth was recorded as (0) absent and (1) present. Teeth were cleaned and dried by sterile gauze and inspected for developmental defects of enamel using disposable dental mirrors. Enamel defects were recorded on the buccal surfaces of each tooth present according to the criteria described by the modified DDE index proposed by FDI, 1992 [1]. Demarcated opacities (coded =1), diffuse opacities (coded= 2), demarcated and diffuse opacities (coded =3), hypoplasia (coded=4) and hypoplasia and opacities (coded =5). Defects measuring less than 1 mm in diameter were excluded and where any doubt exists concerning the presence of a defect, the tooth surface was scored as normal. At the individual level, dummy variables were constructed in terms of DDE=0 (normal) and DDE>1 (presence of demarcated opacity, diffuse opacity or hypoplasia). Dummy variables were also constructed for demarcated opacities, diffuse opacities and hypoplasia in terms of; (0) absent and (1) present, respectively.

### Statistical analyses

Predictive Analytics SoftWare, IBM SPSS Statistics, version 18 was used for data analysis. Univariate analyses were performed by use of chi-square statistics. A probability value of p<0.05 was considered statistically significant. Step wise multiple variable logistic regression analyses with odds ratios (OR) and 95% confidence intervals (CI) were used to identify early life course determinants of DDE and enamel hypoplasia. Moreover, Poisson regression with robust variance, rate ratios (RR) and 95% CI was calculated. Since using dummy variables run the risk of losing information, results from logistic regression analyses were checked using Poisson regression with count variables.

## Results

### Sample characteristics and descriptive analyses

A total of 1221 (99% mothers, mean age 28.3 years, standard deviation 6.5) caregiver/child pairs participated in this study corresponding to a response rate of 97.7%. Totals of 49.1% of the children investigated were females and the mean age was 18.4 months (sd 7.7). About 18% had a history of low birth weight and 60% were currently breastfed. Table 1 depicts the frequency distribution of participants by socio-behavioral and developmental characteristics. About 71% of participating mothers reported at least primary education, whereas one third was below 24 years of age. About 23% of mothers belonged to the poorest household quartile. About 60% of children had visible plaque on upper anterior teeth.

**Table 1:** The frequency distribution of socio-behavioral characteristics

**Variables**	**Categories**	**% (n)**
Sex	Male	50.5 (616)
	Female	49.5 (605)
Number of children/mother	1- 3 children	55.4 (676)
	4 and above children	44.6 (545)
Child age	6-12 months	29.6 (362)
	13-24 months	50.9 (621)
	25-36 months	19.5 (238)
Presence of visible plaque	No	40.1 (490)
	Yes	59.9 (731)
Illness episodes	Few	23.6 (288)
	Many	76.4 (933)
Mother’s perception on child size at birth	Smaller	15.3 (187)
	Average	73.0 (676)
	Larger	11.7 (143)
Breast feeding	No	39.8 (486)
	Yes	60.2 (735)
Mothers education	No formal education	28.2 (344)
	Completed primary and above	71.8 (877)
Mother’s age	≤ 24 years	33.8 (403)
	≥25 years	66.2 (789)
Birth weight	Low (less than 2500gm)	17.9 (50)
	Normal (equal or more than 2500gm)	82.1 (230)
Household assets index	1^st^ quartile-least poor	26.8 (327)
	2^nd^ quartile	25.0 (305)
	3^rd^ quartile	24.8 (303)
	4^th^ quartile- poorest	23.4 (286)

### Reliability and frequency of DDE

**Figure 1:**
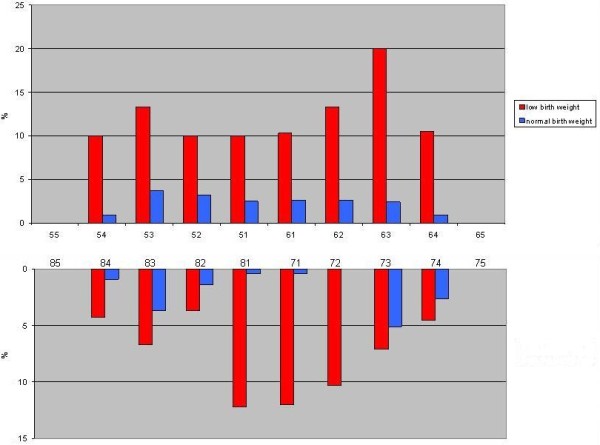
Frequency distribution of enamel hypoplasia according to tooth type in low and normal birth weight children.

To avoid inter examiner inconsistencies, clinical examination was carried out by one trained and calibrated dentist (RM). The calibration for scoring all types of developmental defects (DDE) was conducted with photographs of the DDE index (FDI, 1992) and, the agreement between examiner and the gold standard amounted to Cohen’s kappa 0.82. During the field work, duplicate examinations 3 weeks apart were performed with 80 child-caregiver pairs randomly chosen. Intra examiner reliability in terms of Cohen’s kappa for enamel hypoplasia on the tooth level ranged from 0.91 to 0.97, respectively. Test-re test was not performed for demarcated opacity and diffuse opacity. The total prevalence of enamel defects (DDE >0) amounted to 33.3%. As shown in Table 2, the most common type of defect was diffuse opacity (23.1%), followed by enamel hypoplasia (7.6%) and demarcated opacity (5.0%). Regarding enamel hypoplasia, most children presented with three or more teeth being affected. Table 3 depicts the developmental defects of enamel according to the type of tooth examined. Demarcated opacities were most frequently observed in the central incisors of the upper jaw (2.3%-3.5%). Diffuse opacities were most and least frequently observed in the upper central incisors (24%) and lower central incisors (2.3-2.4%). Hypoplasia was most frequently observed in the upper canines (5%) and least frequently observed in lower central incisors (1%) (Table 3). Figure 1 shows the frequency distribution of enamel hypoplasia according to tooth type in low and normal birth weight children for the 280 children birth weight was accessible from birth certificate or immunization card. Low birth weight with children were most frequently affected across all teeth. In the low birth weight group, the upper left canine was the most- and mandibular right lateral were least frequently affected. Corresponding figures for the normal birth weight group showed that mandibular left canines were most frequently affected and mandibular central incisor the tooth least frequently affected.

**Table 2:** Percentage distribution (n) and number of teeth affected by enamel defects

	**Demarcated opacity**	**Diffuse opacity**	**Hypoplasia**	**DDE**
	**% (n)**	**% (n)**	**% (n)**	**% (n)**
No such defects	95.0 (1160)	76.9 (939)	92.1 (1124)	66.7 (814)
Number of teeth affected				
1 tooth	2.7 (33)	0.9 (11)	1.7 (21)	4.7 (57)
2 teeth	2.0 (24)	12.4 (151)	1.9 (24)	15.2 (186)
≥ 3 teeth	0.3 (4)	9.8 (120)	4.3 (52)	13.4 (164)

**Table 3:** Distribution of types of developmental defects of enamel (DDE) according to tooth type (n=1221)

**Tooth**	**55**	**54**	**53**	**52**	**51**	**61**	**62**	**63**	**64**	**65**
	***% (n)***	***% (n)***	***% (n)***	***% (n)***	***% (n)***	***% (n)***	***% (n)***	***% (n)***	***% (n)***	***% (n)***
*Type of defect*										
Normal	89.7(200)	90.7(485)	87.1(330)	88.2(696)	70.9(713)	69.3(692)	88.0(690)	86.5(326)	89.1(476)	89.1(197)
Demarcated opacities	0.0(0)	0.0(0)	0.0(0)	0.9(7)	2.3(23)	3.5(35)	0.3(2)	0.0(0)	0.0(0)	0.0(0)
Diffuse opacities	8.5(19)	7.1(38)	7.7(29)	8.9(70)	24.4(245)	24.6(246)	9.1(71)	8.5(32)	8.2(44)	8.6(19)
Hypoplasia	1.8(4)	2.2(12)	5.3(20)	2.0(16)	2.3(23)	2.4(24)	2.7(21)	5.0(19)	2.6(14)	2.3(5)
Total	100(223)	100(535)	100(379)	100(789)	100(1004)	100(997)	100(784)	100(377)	100(534)	100(221)
**Tooth**	**85**	**84**	**83**	**82**	**81**	**71**	**72**	**73**	**74**	**75**
	***% (n)***	***% (n)***	***% (n)***	***% (n)***	***% (n)***	***% (n)***	***% (n)***	***% (n)***	***% (n)***	***% (n)***
*Type of defect*										
Normal	89.6(224)	90.0(479)	85.4(310)	95.4(661)	95.8(1162)	95.6(1158)	94.9(654)	86.2(306)	88.8(478)	90.4(225)
Demarcated opacities	0.0(0)	0.0(0)	0.3(1)	0.2(2)	0.9(10)	0.9(10)	0.2(2)	0.6(2)	0.2(1)	0.0(0)
Diffuse opacities	7.6(19)	7.0(37)	7.7(28)	2.5(17)	2.3(28)	2.4(29)	2.9(20)	7.9(28)	8.2(44)	7.2(18)
Hypoplasia	2.8(7)	3.0(16)	6.6(24)	1.9(13)	1.1(13)	1.2(14)	1.9(13)	5.4(19)	2.8(15)	2.4(6)
Total	100(250)	100(532)	100(363)	100(693)	100(1213)	100(1211)	100(689)	100(355)	100(538)	100(249)

### Covariates of DDE

According to unadjusted analyses, sex and age of child, perceived size of child at birth, household assets, current breast feeding and presence of visible plaque in upper anterior teeth were statistically significantly associated with enamel defects (Table 4). Moreover, sex, age of child, presence of visible plaque in upper anterior teeth, perceived child size at birth and current breastfeeding were statistically significantly associated with diffuse opacity. Age of child, perceived child size at birth, current breast feeding and presence of visible plaque were statistically significantly associated with enamel hypoplasia. Breastfeeding and age of child were statistically significantly associated with demarcated opacity (p<0.001).

**Table 4:** Distribution of all types of DDE, separately diffuse opacities, hypoplasia and demarcated opacities according to socio-demographics

		**DDE % (n)**	**Diffuse opacities % (n)**	**Enamel hypoplasia % (n)**	**Demarcated opacities % (n)**
Sex	Male	36.0 (222)*	25.6 (158)*	7.5 (46)	5.7 (35)
	Female	31.0 (188)	20.7 (125)	8.4 (51)	4.3 (26)
Number of children	1- 3 children	34.0 (230)	24.1 (163)	7.8 (53)	3.8 (26)
	4 and above children	33.0 (180)	22.0 (120)	8.1 (44)	6.4 (35)
Child age	6-12 months	20.2 (73)**	9.9 (36)**	3.6 (13)**	7.2 (26)*
	13-24 months	34.0 (211)	25.6 (159)	6.0 (37)	4.7 (29)
	25-36 months	52.9 (126)	37.0 (88)	19.7 (47)	2.5 (6)
^a^ Presence of visible plaque	No	21.4 (105)**	12.4 (61)**	3.7 (18)**	5.9 (29)
	Yes	41.7 (305)	30.4 (222)	10.8 (79)	4.4 (32)
Illness episode	Few	35.1 (101)	24.7 (71)	4.5 (13)**	6.6 (19)
	Many	33.1 (309)	22.7 (212)	9.0 (84)	4.5 (42)
Birth weight	less than 2500 gm	36.0 (18)	12.0 (6)	22.0 (11)**	2.0 (1)
	Equal or more than 2500 gm	33.0 (76)	23.5 (54)	7.4 (17)	3.5 (8)
Mother’s perception on child size at birth	Smaller	42.2 (79)**	31.0 (58)*	12.3 (23)**	3.2 (6)
	Average	31.5 (281)	21.9 (195)	6.4 (57)	5.6 (50)
	Larger	35.0 (50)	21.0 (30)	11.9 (17)	3.5 (5)
Breast feeding	No	43.0 (209)**	31.5 (153)**	13.4 (65)**	2.7 (13)**
	Yes	27.3 (201)	17.7 (130)	4.4 (32)	6.5 (48)
Mothers education	No formal education	34.0 (117)	22.1 (76)	9.3 (32)	5.5 (19)
	Completed primary and above	33.4 (293)	23.6 (207)	7.4 (65)	4.8 (42)
Mother’s age	≤ 24 years	31.8 (128)	21.8 (88)	6.9 (28)	4.7 (19)
	≥25 years	35.1 (277)	24.1 (190)	8.7 (69)	5.3 (42)
Household assets index	1^st^ quartile-least poor	27.8 (91)*	19.3 (63)	7.0 (23)	4.0 (13)
	2^nd^ quartile	34.8 (106)	24.3 (74)	6.6 (20)	5.9 (18)
	3^rd^ quartile	35.0 (106)	25.4 (77)	7.9 (24)	5.0 (15)
	4^th^ quartile- poorest	37.4 (107)	24.1 (69)	10.5 (30)	5.2 (15)

P<0.05, **P<0.01, ^a^ presence of visible plaque on upper anterior teeth.

All socio-demographic-, behavioral- and developmental variables that were statistically significantly associated with DDE and hypoplasia in the bivariate unadjusted analyses (Table 4) were included into multivariable logistic regression analyses and Poisson regression analyses. The variables entered into multivariable analyses were selected from those reported to have an association with DDE in previous studies [13]. They were entered into the regression model following the conceptual framework proposed by Zhou et al. [8]. According to the theoretical model, early level 1 life course factors in terms of socio-economic position, child illness episodes, perceived size of child at birth and birth weight were entered into the first step of the multivariable models. Subsequent level 2 and 3 life course factors in terms of current breastfeeding and current oral hygiene (visible plaque) were entered into step II and III, respectively. As shown in Table 5, the final logistic regression model with respect to enamel defects showed that children belonging to the older age groups were associated with higher odds of having DDE [OR 4.1 (95% CI 1.3 - 12.8)]. A female child was associated with lower odds of having enamel defects [OR 0.5 (95% CI 0.2 -0.8)]. According to Table 6, the final logistic regression model with respect to hypoplasia revealed that belonging to the normal birth weight group (equal or more than 2500 g) associated with lower odds of having enamel hypoplasia [OR 0.2 (95% CI 0.1-0.7)]. Breastfeeding status and presence of visible plaque in upper anterior teeth did not maintain statistical significance in the multiple variable analyses. Poisson regression confirmed the results from multiple variable logistic regression analyses presented in Tables 5 and 6.

**Table 5:** Developmental enamel defects, DDE, regressed on early and current life course factors

		**Logistic regression**			**Poisson**
		**Step I**	**Step II**	**Step III**	
		**Nagelkerkes R**^**2 **^**= 0.167**	**Nagelkerkes R**^**2 **^**= 0.168**	**Nagelkerkes R**^**2 **^**= 0.182**	**Adjusted RR (95% CI)**^**c**^
		**OR (95% CI)**	**OR (95% CI)**	**OR (95% CI)**	
**Level I:**					
Sex	Male	1.0	1.0	1.0	1.4(1.07-2.03)
	Female	0.5(0.2- 0.8)	0.5(0.2- 0.8)	0.5(0.2- 0.8)	1.0
Child age	6-12 months	1.0	1.0	1.0	0.4(0.2-0.9)
	13-24 months	2.3(1.1- 4.8)	2.1(0.9- 4.6)	1.6(0.6- 3.7)	0.6(0.4-0.9)
	25-36 months	7.3(3.3-16.1)	5.9(2.1- 17.1)	4.1(1.3- 12.8)	1.0
Mother’s perception on child size at birth	Smaller	1.0	1.0	1.0	0.7(0.4-1.4)
	Average	1.1(0.5-2.4)	1.1(0.4- 2.5)	1.1(0.4- 2.5)	0.7(0.5-1.3)
	Larger	1.6(0.5- 5.3)	1.6(0.5- 5.5)	1.7(0.5- 5.8)	1.0
Household assets index	1^st^ quartile-least poor	1.0	1.0	1.0	0.9(0.6-1.5)
	2^nd^ quartile	0.7(0.4- 1.5)	0.7(0.4- 1.5)	0.7(0.3- 1.4)	0.7(0.5-1.2)
	3^rd^ quartile	0.7(0.3- 1.6)	0.7(0.3- 1.6)	0.7(0.3- 1.5)	0.7(0.5-1.3)
	4^th^ quartile- poorest	1.1(0.5- 2.5)	1.1(0.5- 2.5)	1.1(0.5- 2.5)	1.0
Birth weight	Less than 2500 g	1.0	1.0	1.0	1.1(0.72-1.71)
	More than 2500 g	0.8(0.4- 1.9)	0.8(0.4- 1.9)	0.8(0.4- 1.8)	1.0
**Level II:**					
Breast feeding	No		1.0	1.0	1.0(0.7-1.7)
	Yes		0.8(0.4- 1.6)	0.9(0.4- 1.9)	1.0
**Level III:**					
Presence of visible plaque	No			1.0	0.6(0.3-1.2)
	Yes			1.9(0.9- 4.1)	1.0

^c^: reference category: the last category by default.

**Table 6:** Enamel hypoplasia regressed on early- and current life course factors

		**Logistic regression**			**Poisson**
		**Step I**	**Step II**	**Step III**	
		**Nagelkerkes R**^**2 **^**= 0.224**	**Nagelkerkes R**^**2 **^**=0.239**	**Nagelkerkes R**^**2 **^**= 0.257**	**Adjusted RR (95% CI)**^**c**^
		**OR (95% CI)**	**OR (95% CI)**	**OR (95% CI)**	
Level I					
Sex	Male	1.0	1.0	1.0	1.8(0.9-3.6)
	Female	0.4(0.2-1.1)	0.4(0.2-1.1)	0.4(0.2-1.1)	1.0
Child age	6-12 months	1.0	1.0	1.0	1.0(0.2-4.8)
	13-24 months	1.4(0.3-5.1)	0.7(0.1-3.8)	0.4(0.1-2.6)	0.5(0.2-1.3)
	25-36 months	6.5(1.8-23.0)	2.2(0.3-14.8)	1.1(0.1-8.9)	1.0
Mother’s perception on child size at birth	Smaller	1.0	1.0	1.0	1.1(0.3-3.4)
	Average	0.3(0.1-1.1)	0.3(0.1-1.2)	0.3(0.1-1.2)	0.4(0.2-1.1)
	Larger	0.8(0.1-4.0)	0.9(0.1-4.7)	1.0(0.2-5.2)	1.0
Household assets index	1^st^ quartile-least poor	1.0	1.0	1.0	0.9(0.3-2.5)
	2^nd^ quartile	0.8(0.2-2.6)	0.8(0.2-2.6)	0.7(0.2-2.4)	0.7(0.2-1.9)
	3^rd^ quartile	0.8(0.3-2.8)	0.8(0.2-2.7)	0.7(0.2-2.6)	0.7(0.2-2.2)
	4^th^ quartile- poorest	1.2(0.3-4.4)	1.2(0.3-4.3)	1.1(0.2-4.1)	1.0
Illness episode	Few	1.0	1.0	1.0	0.7(0.2-2.1)
	Many	1.4(0.4-4.5)	1.5(0.5-4.9)	1.5(0.5-5.1)	1.0
Birth weight	less than 2500 g	1.0	1.0	1.0	2.9(1.4-6.1)
	More than 2500 g	0.2(0.1-0.7)	0.2(0.1-0.8)	0.2(0.1-0.7)	1.0
Level II					
Breast feeding	No		1.0	1.0	2.2(0.6-8.1)
	Yes		0.3(0.7-1.5)	0.4(0.1-1.8)	1.0
Level III					
Presence of visible plaque	No			1.0	0.3(0.1-1.6)
	Yes			3.3(0.7-15.0)	1.0

^c^: reference category: the last category by default.

## Discussion

There is a lack of population based studies emanating from developing countries that consider developmental enamel defects in the primary dentition. To our knowledge this study is the first to estimate the frequency and early life-course determinants of enamel defects among 6–36 months old children attending RCH clinics for growth monitoring and immunization purposes in northern Tanzania. A substantial frequency of total enamel defects, amounting to 33%, was estimated. This frequency falls within the range of 24-49% reported in the primary dentition of children in developed countries [12,13]. Notably, however, the frequency observed in this study population might be an underestimation as long as only buccal surfaces were recorded and due to the young age of the children investigated. Thus, comparisons of prevalence estimates with other studies should be made with caution since age groups investigated and diagnostic criteria implemented may have varied across the studies. Nevertheless, the present frequency accords with that of Seow et al. [4] who reported on a prevalence of 25% in the primary dentition of Australian children living in low fluoride districts, but is far below that reported from indigenous communities in Australia [28]. In Brazil, a birth cohort study following children to the age of 54 months revealed that 81.3% presented with at least one tooth affected with DDE [29]. Even higher rates of enamel defects amounting to 70-80% have been presented by Cruvinel et al. [21] and by Chaves et al. [18]. On the other hand, the frequency observed in this study is far above what has been reported among preschool children from various regions in Tanzania [7]. In accordance with some previous studies but at odds with others, diffuse opacities were the most common defects identified in this study (23.1%), followed by hypoplasia (7.6%) and demarcated opacities (5.0%) [18]. In contrast, Seow et al. [4] found a relatively low prevalence of diffuse opacities in the primary dentition of Australian children from low fluoride communities. Correa-Faria et al. [13] observed that demarcated opacities were the most prevalent defect in the primary dentition of Brazilian children. Consistent with previous studies [21], the groups of teeth most frequently affected in Manyara children 6–36 months old were in descending order incisors, canines and molars, whereas hypoplasia was most common in upper canines and least common in lower incisors (Table 3). The distribution of DDE according to tooth type should be interpreted with caution due to the fact that only a partially erupted dentition was examined. Evidence suggests that among the different types of enamel defects, hypoplasia is the defect most frequently associated with ECC [7]. Thus, the prevalence of hypoplasia observed in this study is likely to contribute to an increased risk of future caries [30]. A significant and positive association between enamel hypoplasia and ECC has been reported previously among the participants of this study [31], suggesting that enamel defects could be included as a dental health indicator in epidemiological studies of children in Tanzania.

It should be noted that although there is multiple factors that may cause enamel defects, its clinical appearance is often non-specific leading to difficulties in the diagnosis. Thus, diffuse opacities of enamel are the feature distinguishing the teeth of children living in low and high fluoridated areas [32]. The level of natural fluoride is high in the north eastern part of Tanzania. However, the exact values for the Manyara region are still unknown, although the study site is part of this high fluoridated area. It is plausible that diffuse opacities observed in this study might be attributed to high fluoride levels in the drinking water. In contrast to the permanent dentition, where diffuse opacities are the most commonly found enamel defect in communities exposed to optimum ranges of fluoride in drinking water, the primary dentition is assumed to be less affected by fluorosis as the fetus is generally protected in utero from excessive fluoride levels, and by being breastfeed after birth [33]. Nevertheless, in this study, enamel defects were assessed using the modified DDE index that is based on the premise that the etiology should not be presumed [34]. It may be questioned whether white spot early caries lesions have been misclassified as enamel opacities. However, these lesions are usually easily differentiated since white spot caries are placed adjacent to the gingival margin and extends along the labial and lingual surfaces. In contrast, developmental opacities have no preferential location on the tooth. Moreover, caries lesions may have masked pre-existing enamel defects and confused diagnosis. Difficulties in discriminating between enamel hypoplasia and cavities with arrested caries may have led to an overestimation of enamel defects. To limit the possibility of misclassifications, the dental recorder in this study was calibrated according to the guidelines published by British Association of the study of Community Dentistry (BASCD) [27]. Moreover, children were examined carefully in knee to knee position using a dental mirror and their teeth were cleaned and dried by sterile gauze before being examined with respect to ECC and DDE.

Socio-economic status of the family and early childhood infectious diseases have been associated with increased enamel defects in the primary dentition [17,18,35]. Such relationships were indicated in unadjusted analysis but did not remain statistically significant in the fully adjusted multivariable models (Tables 4, 5 and 6). Nevertheless, older children and girls were respectively more and less likely to present with developmental defects than their younger counterparts and boys, independent of all other factors considered (Table 5). This result is contrary to some studies [21] but is accordant with findings reported by Li et al. [35]. It has been suggested that increased enamel defects in males have been caused by increased nutritional requirements due to more rapid growth thus making males more susceptible than females to the formation of enamel defects. Assessing socio-economic status in terms of international classification of occupational status is not easily applied in developing countries although modifications have been proposed [25]. Unadjusted analyses revealed that children with presence of dental plaque, indicating poor oral hygiene, were more likely to present with enamel defects than their counterparts with less observable plaque (Table 4). Poor oral hygiene may be attributed to deprivation in general. It is also plausible that increased sensitivity in teeth with DDE might interfere with caretaker’s tooth cleaning procedures. Children currently not breastfed and children with many episodes of early childhood illness were most likely to present with all types of enamel defects, but only in the bivariate analyses. It is probable that the association between breastfeeding and enamel defects was confounded by child’s age as current breastfeeding was most frequent among the younger children, whereas enamel defects increased with children’s age. Malnutrition is a phenomena of poverty and the prevalence of stunting in Tanzanian children less than 5 years have been estimated to 25% in urban and 45% in rural areas [36]. Since enamel of primary teeth is approximately completed during the first year of life, it is nutritional disturbances during the neonatal period that most probably might cause enamel defects in the primary dentition [37]. Prematurely born children (< 37 weeks gestation) have shown an increased prevalence of DDE with incidences amounting to 96% [15]. Accordingly, in this study the prevalence of hypoplasia, was higher among children with a history of low birth weight (<2500 g) assuming that low birth weight reflects both poor nutritional status during pregnancy and or prematurity. Moreover, the present finding is supported by previous ones, where significant effects of low birth weight have been reported for hypoplasia alone and not for the other types of enamel defects [21,35]. As shown in Table 6, normal birth weight children were less likely to develop DDE after having adjusted for other early- and more current life course factors [23]. In the present study the prevalence of enamel hypoplasia in low birth weight children was about 22%. Others have reported higher prevalence of DDE ranging from 51% to 96% [15].

In spite of some limitations of this study, such as use of very few early life events and a cross-sectional design, the latter making conclusions about causal effect impossible and a reversed causality an option, there are strengths to emphasize. Data on birth weight was taken from the birth certificates, thus avoiding bias related to parental self-reported information and recall. Notably, comparisons of the results with other studies available in the literature must be done with caution due to differences in sample delineation, environmental influences (fluoride) and methodologies. Maternal disorders recognized to be the primary causes of prematurity and low birth weight, such as hypertension, preeclampsia, gestational diabetes and cardiopathy were not considered in the present study. Since only 22.9% of the primary caretakers made the birth card available – this loss might have resulted in biased estimates of the association between birth weight and enamel defects. The fact that the oral examination was not performed in a dental clinic may have reduced the true frequency of DDE and the tooth drying technique utilized may have confounded opacities and white spots caused by demineralization.

## Conclusion

Considering the methodology of this study it can be concluded that there was a moderately high frequency of enamel defects in the total sample that increased with age and was less common in girls than in boys. Most developmental defects observed presented as diffuse opacities, whereas the frequency of hypoplastic enamel defects was less substantial. However, low birth weight children had a higher risk of presenting with hypoplastic defects compared with their normal birth weight counterparts. In view of the frequent occurrence of DDE and the fact that enamel hypoplasia constitutes a risk factor for future ECC, enamel defects should be included as a dental health indicator in epidemiological studies of children in north eastern Tanzania.

## Competing interests

The authors declare that they have no competing interests.

## Authors’ contributions

RM: principal investigator, designed the study, collected the data, performed the statistical analyses, and wrote the Manuscript. AB: participated in the design of the study and provide valuable guidance in the data collection and has been actively involved statistical analyses and writing the manuscript. AN: main supervisor, designed the study, guided the statistical analyses and writing the manuscript. All authors have read and approved the final manuscript.

## Authors’ information

RM: PhD candidate, University of Bergen. AB: Professor, Department of Clinical Dentistry, University of Bergen, Norway. AN: DDS PhD, Professor, Department of Clinical Dentistry, Community Dentistry, and Centre for International Health, University of Bergen, Norway.

## Pre-publication history

The pre-publication history for this paper can be accessed here:

http://www.biomedcentral.com/1472-6831/13/21/prepub
